# Regulating Cdc42 and Its Signaling Pathways in Cancer: Small Molecules and MicroRNA as New Treatment Candidates

**DOI:** 10.3390/molecules23040787

**Published:** 2018-03-29

**Authors:** Xing-Hua Xiao, Lin-Chen Lv, Jing Duan, Ye-Meng Wu, Shu-Jin He, Zhen-Zhen Hu, Li-Xia Xiong

**Affiliations:** 1 Department of Pathophysiology, Medical College, Nanchang University, 461 Bayi Road, Nanchang 330006, China; xiaoxinghua1988@sohu.com (X.-H.X.); l.lyu@se15.qmul.ac.uk (L.-C.L.); qbdlxx@163.com (J.D.); 18279146700@163.com (Y.-M.W.); 13699532409@126.com (S.-J.H.); 2 Jiangxi Province Key Laboratory of Tumor Pathogenesis and Molecular Pathology, 461 Bayi Road, Nanchang 330006, China

**Keywords:** Cdc42, Cdc42-related signaling pathways, small molecules, miRNAs, cancer therapy

## Abstract

Despite great improvements in the diagnosis and treatment of neoplasms, metastatic disease is still the leading cause of death in cancer patients, with mortality rates still rising. Given this background, new ways to treat cancer will be important for development of improved cancer control strategies. Cdc42 is a member of the Rho GTPase family and plays an important role in cell-to-cell adhesion, formation of cytoskeletal structures, and cell cycle regulation. It thus influences cellular proliferation, transformation, and homeostasis, as well as the cellular migration and invasion processes underlying tumor formation. Cdc42 acts as a collection point for signal transduction and regulates multiple signaling pathways. Moreover, recent studies show that in most human cancers Cdc42 is abnormally expressed and promoting neoplastic growth and metastasis. Regarding possible new treatments for cancer, miRNA and small molecules targeting Cdc42 and related pathways have been recently found to be effective on cancer. In this review, we analyze the newly recognized regulation mechanisms for Cdc42 and Cdc42-related signal pathways, and particularly new treatments using small molecules and miRNAs to inhibit the abnormal overexpression of Cdc42 that may slow down the metastasis process, improve cancer therapy and lead to novel strategies for development of antineoplastic drugs.

## 1. Introduction

**Figure 1:**
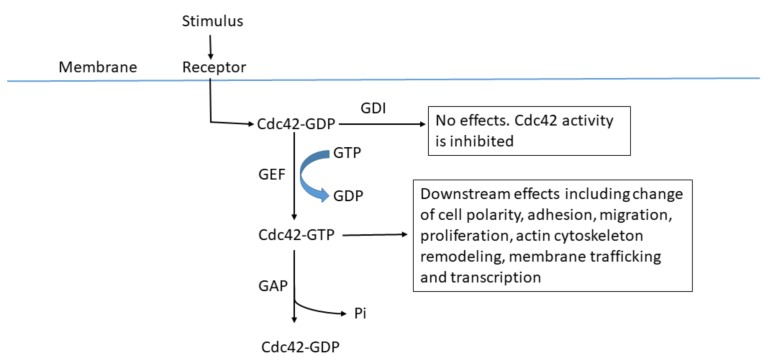
Regulation of Cdc42. Cdc42 switches between an inactive GDP-bound state and an active GTP-bound state. The GTP binding and hydrolysis cycle is highly regulated by intracellular molecules GEFs, GAPs, and GDIs. When Cdc42 is activated by various stimuli, Cdc42 can transiently interact with its downstream effector proteins, triggering cytoskeleton reorganization, alterations in the cell cycle, and transcription.

Cell division control protein 42 homolog (Cdc42) is a member of the Rho GTPase family, which was first discovered in *Saccharomyces cerevisiae* cells, where it was found to be a marker of the loci where new buds would appear. Earlier in vivo studies in mammals indicated that Cdc42 plays an important role in the regulation of the cardiovascular system, immune system, nervous system, and bone modeling. Cdc42 is also essential for the development of the pancreas, blood cells, eyes, and skin [1]. The Cdc42 active molecular switch is opened in the GTP-bound state and closed in the GDP-bound state, which is regulated by the guanine nucleotide exchange factors (GEFs), GTPase activating proteins (GAPs), and guanine nucleotide dissociation inhibitors (GDIs). GEFs catalyze the exchange from GDP- to GTP-activating Cdc42 that can then activate downstream effectors, leading to altered cellular proliferation, polarity, adhesion, and migration, as well as dynamic changes of the cytoskeleton and other cell functions [2]. Conversely, GAPs play a negative regulatory role on the activity of Cdc42 via hydrolysis of GTP to GDP; GDIs lock the GDP-bound inactive state of Cdc42 and prevent its further activation [3]. This is the classic “GTPase cycle” model of Cdc42 (Figure 1). Based on this mechanism, many small molecule inhibitors have been developed that are targeted to Cdc42 and its signaling proteins [4].

Several recent studies have revealed unconventional mechanisms regulating the Rho GTPase signaling activity. For example, microRNAs regulate post-transcriptional processing of Rho GTPase-encoding mRNAs. Palmitoylation and nuclear translocation signals have an effect on intracellular distribution and post-translational phosphorylation. Some micro-RNAs can regulate the expression of Cdc42 through directly binding to 3’UTR of Cdc42 mRNA, which changes the activity of downstream effector/adaptor proteins of Cdc42 [5]. Transglutamination and AMPylation also affect Rho GTPase signaling, and the stability and turnover of the Rho GTPase protein is controlled by ubiquitination [5]. 

This review will address the post-transcriptional regulation of Cdc42 by microRNAs. We will describe the expression and regulators of Cdc42 and discuss their downstream effectors in cancers. Finally, we will summarize the small molecule inhibitors and micro-RNAs regulating Cdc42 activity.

## 2. The Expression of Cdc42 in Cancer

According to the Sanger COSMIC database (Catalogue of Somatic Mutations in Cancer), 41 mutations of Cdc42 have been reported in a variety of tumor tissues [6]. However, the frequency of Cdc42 mutations is low, ranging from 0.06 to 0.73%. Copy number variations of Cdc42 are still uncommon (<1% according to COSMIC) and found in only 11 tissues. The G12V variant of Cdc42 was identified in a melanoma biopsy as an oncogenic Ras mutation [6]. Moreover, the expression of the Cdc42 (F28L) mutant promotes cellular growth with low-serum conditions and malignant transformation of NIH 3T3 cell lines [7]. Some Cdc42 mutants can exchange GDP for GTP but still hydrolyze GTP (called ‘fast-cycling’ mutants) promote cellular transformation, whereas Cdc42 mutants that cannot hydrolyze GTP (called ‘slow-cycling’ mutations) and are irreversibly trapped in the GTP-bound state often inhibit cell growth. It has been proved that GAP can reverse the transformation caused by the ‘fast-cycling’ mutants Cdc42[F28L] [8]. In addition, some Cdc42 mutants can change the structure of Cdc42, which may leads to a strong interaction with the effector proteins such as PAKs. Although further molecular researches are needed to confirm this these possibilities and have clearer understanding of the Cdc42 mutant structures [9]. The gene expression of Cdc42 is different in several types of cancer. For instance, its expression is significantly downregulated in central nervous system (−20.09%), adrenal gland (−15.19%), kidney (−11.50%), and pancreatic cancers (−10.61%) [8]. Yet it is upregulated in cancers of the cervix (+8.79%), stomach (+5.61%), esophagus (+5.60%), prostate (+4.82%), lung (+4.13%), hematopoietic/lymphoid tissues (+4.07%), and thyroid gland (+3.31%) [10]. Cdc42 is associated with oncogenesis non-small cell lung cancer (NSCLC), gastric cancer, breast cancer, and squamous cell carcinoma of the esophagus. But Cdc42 may act as an anti-cancer gene in hepatocellular carcinoma. In conclusion, the role of Cdc42 in cancer is diverse and needs further exploration.

## 3. Regulators of Cdc42

### 3.1. Guanine Nucleotide Exchange Factors (GEFs)

**Figure 2:**
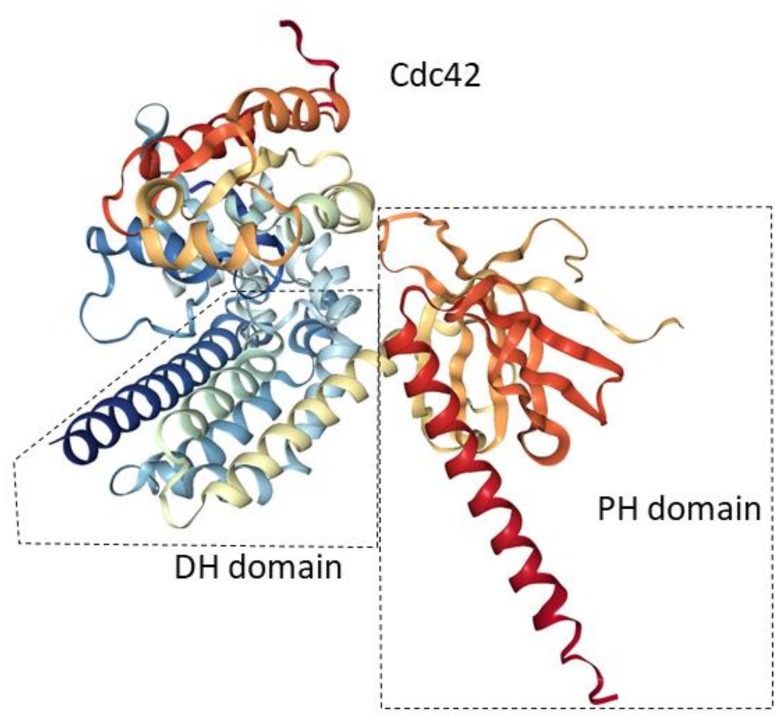
Three-dimensional structures of DH–PH domains. A ribbon diagram shown the DH domain (left dotted area) and PH domain (right dotted area) binding to Cdc42 (upper area). PDB accession number 1KZ7 [15]. GDP-bound Cdc42 (Rho GTPase) favor interactions with GEFs (as indicated by DH and PH in the figure). DH domain is essential for the biological activity of GEFs while PH domain can directly regulate the activity of the DH domain.

Like other GTPases, Cdc42 switches between an inactive GDP-bound state and an active GTP-bound state; this configuration determines whether a downstream effect is produced. GEFs catalyze the exchange of GDP with GTP, which results in the activation of downstream effector proteins that produce key effects. The typical GEF contains a Dbl homology (DH) domain, which is essential for its biological activity [11]. Also, some additional domains such as DOCK180, which mediates interactions between lipids and peptides, have been found to promote the guanine nucleotide exchange of Rac (another member of the small GTPase family) with the DH domain [12]. A Pleck–Strin homology (PH) domain is adjacent to the DH domain that mediates membrane localization and has direct regulatory effects on the DH domain (Figure 2). Comparative analyses of the crystal structure of the DH–PH domains of the ‘son of sevenless’ (Sos) protein GEFs in the presence and absence of the lipid ligand state have shown that the PH domain can affect the conformation of the adjacent DH domain and regulate its activity [13]. Moreover, additional domains specific to each GEF may modulate the mechanisms of the activation and regulation of the GEFs [14]. 

### 3.2. GTPase-Activating Proteins (GAPs)

Conversely, the GAPs function to inactivate Cdc42 by hydrolyzing the GTP to GDP. There are about 20 types of human GAPs, eight of which are coded by loci on chromosome 22, according to the human genome sequencing project. Of these GAPs, Rga1, Rga2, and Bem3 are relatively well-studied. Although they share similar activity of the GAP domain, their protein sequences are not similar [11]. Rga1 contains two tandem Lin-11, Isl-11, Mec-3 (LIM) domains while Bem3 contains a PH domain. Lim domains mediate protein interactions through binding with zinc ions. The function of the PH domain is to play a role in membrane localization. The study of Smith et al. [11] demonstrated that these GAPs for Cdc42 play unique roles in haploid invasive growth and septin organization. Bem3 may play a more crucial role in mediating Cdc42 and Cla4 effects in cytokinesis, while Rga1 and Rga2 largely regulate interactions between Cdc42 and Ste20 but seem to regulate the morphologies of the cells to a lesser extent. Another important discovery is that after the comparison of the crystal structures of the ground-state complex between RhoGAP and Cdc42 guanosine 5’-(β,γ-imido) triphosphate, a 20° rotation between GTPase and GAP leads to an arginine residue of the GAP protein entering the active GTPase site to mediate and stabilize a change from the ground state to a transition state [14].

### 3.3. Guanosine Nucleotide Dissociation Inhibitors (GDIs)

The GDIs comprise a class of GTPase inhibitors that can block GDP-bound Cdc42 in the cytoplasm and inhibit Cdc42 activation. Nuclear magnetic resonance (NMR) images and crystal structures of GDI show that it has an immunoglobulin-like *C*-terminal domain containing a hydrophobic pocket that can accommodate geranyleranyl lipids, and a flexible *N*-terminal domain to inhibit GDP to GTP exchange [14]. GDI competes with the Cdc42 regulator and effector proteins but also blocks G-proteins in the cytoplasm, so that GTPase activity can only be switched on in the absence of GDI. Additionally, the ERM (ezrin, radixin, and moesin) family of proteins can release Rho from RhoGDI and then mediate translocation of Rho from the cytoplasm to the membrane [12,16].

## 4. Downstream Effector/Adaptor Proteins of Cdc42 in Cancer

**Figure 3:**
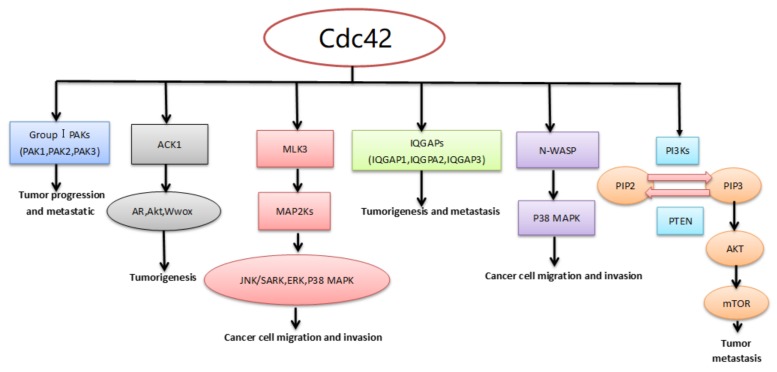
Downstream effector/adaptor proteins of Cdc42. Cdc42 can activate downstream proteins of Cdc42 including PAKs, ACK1, MLK3, IQGAPs, N-WASP, and PI3Ks. Ack1 promotes tumorigenesis by phosphorylating Wwox, Akt, and androgen receptors. PAK1, PAK2, and PAK3 can promote tumor progression and metastasis. MLK3 can phosphorylate and activate specific MAP2Ks, which activate specific MAPKs in sequence. The three major MAPKs are JNK/SAPK, ERK, and P38 MAPK. They can promote cancer cell migration and invasion. IQGAP1 promotes tumorigenesis and metastasis, while IQGAP2 may have the opposite effect. The function of IQGAP3 is not very clear at present. N-WASP can promote cancer cell migration and invasion by phosphorylating P38 MAPK. PIP2 is phosphorylated to PIP3 by PI3K, which leads to the phosphorylation of Akt. And Akt can phosphorylate and activate mTOR, which can promote tumor metastasis. Otherwise, PIP3 is quickly dephosphorylated to PIP2 under the action of PTEN.

Cdc42 is able to activate more than 20 effector/adaptor proteins, including kinases, phospholipases, actin-associated proteins, and others [2]. This section will address the roles in tumors of some protein kinases, such as activated Cdc42 kinase 1 (ACK1), p21-activated kinases (PAKs), mixed-lineage kinase 3 (MLK3), isoleucine-glutamine-motif containing GTPase-activating proteins (IQGAPs), neural Wiskott–Aldrich syndrome proteins (N-WASP), and phosphoinositide 3-kinases (PI3Ks) (Figure 3). 

### 4.1. Activated Cdc42 Kinase 1 (ACK1)

Activated Cdc42 kinase 1 (ACK1), which is also called TNK2, belongs to the Ack family of nonreceptor tyrosine kinases (NRTKs). Structurally, ACK1 has four parts: an *N*-terminal sterile alpha motif (SAM) domain, a SH3 domain, a kinase domain, and a Cdc42/Rac-interactive domain (CRIB). Cdc42 can activate ACK1 by directly and specifically binding to it. ACK1 has been shown to be involved in a variety of cancers, and its overexpression promotes the progression of many of them. In prostate cancer, ACK1 is able to phosphorylate and activate AR function and degrade the phosphorylated tumor suppressor Wwox. Moreover, phosphorylation of Ack1 Tyr284 may serve as a marker of prostate cancer progression [17,18,19]. Activation of Ack1 also plays a crucial role in breast cancer metastasis and is related to poor prognosis [20,21]. However, the influence of Ack1 on cell proliferation has nothing to do with its clinical effects, but rather plays a possible scaffolding function [22,23]. In general, Ack1 exerts effects in cancer cells through both kinase-independent (protein–protein interactions) and kinase-dependent actions. Recent studies have shown that ACK can regulate the proliferation and apoptosis of cells through interaction with the Yorkie (Yki) cofactor. Drosophila ACK, as a Hippo pathway regulator, can promote tissue overgrowth through inhibiting ex-mediated Yki regulation [24].

### 4.2. P21-Activated Kinases (PAKs)

The p21-activated kinases (PAKs) are Ser/Thr kinases. The family of PAKs can be divided into two groups according to structure and function: Group I contains PAK1, PAK2, and PAK3 and Group II contains PAK4, PAK5, and PAK6. Group I is characterized by an auto-inhibition region and a kinase region activated by Cdc42/ Rac1, while Group II lacks these features and cannot be activated by the Rho GTPases. PAK1, as the best characterized member of Group I PAKs, has been shown to act as an oncogene in various tumors. Overexpression of PAK1 has been correlated with NSCLC [25], breast cancer, prostate cancer [26], pancreatic cancer, gastric cancer [27,28], colorectal carcinoma [29], and oral cancers [30]. Moreover, it has been reported that the PAK1-deficient mutant (RB689) of *C. elegans* has a 60% longer lifespan than the wild type, indicating that PAK1 may shorten the lifespan [31], and PAK1-deficient mutant mice appear to be healthier than the wild type [32]. PAK1 has been studied extensively, its association with tumors has been confirmed; in contrast, there have been few similar reports involving PAK2 and PAK3. The expression of PAK2 is upregulated in gastric cancer and is associated with an unfavorable prognosis [33]. Deng et al. [34] found that PAK2 is highly expressed in adenoid cystic carcinoma and promotes cellular migration and proliferation. Inhibition and knockdown of PAK2 can reduce migration of ovarian cancer cells [35]. In addition, recent research shows that PAK3 and SGK2 play key roles in the survival of HPV+ cervical cancer cells, while the lethality of PAK3 and SGK2 shRNAs for HPV+ cervical cancer cells has nothing to do with suppressing PAK3 or SGK2 expression but likely is exerted through non-specific RNAi effects [36].

The PAK signaling pathway is involved in multiple human malignancies including lung, liver, colon, and breast cancers. PAK1 overexpression occurs in 75% of hepatocellular carcinoma tissues, and it results in increased invasiveness, poorer cellular differentiation, uncontrolled neoplastic survival, and tumor growth [37]. Other molecules are also involved in the abnormal overexpression of PAKs, including cyclin D1, Ras, Rac and ERK1/2 [38]. However, drugs targeting PAKs and other molecules in the PAK signaling pathways, such as PF-3758309, FL172, IPA-3, and LCH-7749944, only theoretically inhibit PAK-overexpression-induced cancers [39,40,41,42,43]. Many obstacles such as cytotoxicity, competition for ATP, and failed in vivo tests need to be overcome to improve the efficacy of the small molecule PAK inhibitors.

### 4.3. Mixed-Lineage Kinase 3 (MLK3)

Mixed-lineage kinase 3 (MLK3), also named MAP3K11, is one member of the MLK family. The MLK family has some common structures: an amino *N*-terminal Src-homology 3 (SH3) domain, a catalytic domain, leucine/isoleucine zipper (LZ) motifs, and a CDC42-/Rac1-interactive binding (CRIB) motif. MLK3 acts as a MAP3K that phosphorylates and activates specific MAP2Ks, which activate specific MAPKs in sequence. The sequence of the three major MAPKs is c-Jun *N*-terminal kinase (JNK)/stress-activated protein kinase (SAPK), extracellular-signaling regulated kinase (ERK), and P38 MAPK. The combination of GTP-bound Cdc42 with MLK3 can disrupt SH3-mediated autoinhibition through its CRIB motif, leading to conformational changes that phosphorylate and activate MLK3. Cdc42 activates MLK3 by inducing its homo-dimerization and autophosphorylation, which in turn activate its downstream signaling effectors [42]. The MLK3-JNK signaling axis plays an important role in cell migration in breast cancers. Ectopic expression of MLK3 can induce cell migration whether immortalized breast epithelial cells or poorly invasive breast cancer cells are present, while inhibition of the MLK3 or JNK pathways can block the migration of TNBC cells [44]. MLK3 is essential for FRA-1 expression in breast cancer cells and raises MMP-1 and MMP-9 by FRA-1, which is important for breast cancer cell invasion and transendothelial migration [45]. MLK3 can also promote cell invasion in other cancers, such as NSCLC [46], breast cancer, melanoma, and ovarian cancer [47,48].

### 4.4. Isoleucine-Glutamine-Motif Containing GTPase-Activating Proteins (IQGAPS)

The IQGAP proteins are so-named because they have two key regions: a calmodulin binding IQ-motif and a GTPase activating protein (GAP) related domain (GRD). There are three IQGAP isoforms in humans: IQGAP1, IQGAP2, and IQGAP3. They have some common structural domains: the actin-binding calmodulin homology domain (CHD), the IQ-motif, and the GRD [49]. GTP-Cdc42 and GTP-Rac1 can activate IQGAP1 and IQGAP3 by binding to their GRD, while IQGAP2 is able to interact with the GDP- and GTP-bound forms [49,50]. IQGAP1 acts as an oncogene that promotes tumorigenesis and metastasis, while IQGAP2 may have the opposite effect. However, IQGAP3’s function is not very clear and there are few relevant reports. The level expression of IQGAP1 is elevated in many types of cancers, including NSCLC [51], colorectal carcinoma [52], hepatocellular carcinoma [53], thyroid cancer [54], and pancreatic cancer [55]. IQGAP1 plays an important role in cell proliferation, transformation, migration, cell-cell adhesion, and exocytosis through binding to partners including components of the MAPK cascade, β-catenin, Rac1 and Cdc42, E-cadherin, actin, calmodulin, APC, and others [49]. The absence of IQGAP2 in SV129J mice resulted in IQGAP1-dependent development of HCC at about 12 months [56]. Both the IQGAP2 mRNA and IQGAP2 protein levels are downregulated in human hepatocellular carcinoma (HCC) [53]. In addition, the expression of IQGPA2 is decreased in hormone-refractory prostate cancer [57]. Therefore, IQGAP2 may act as a tumor suppressor. Recently, Xu et al. have found that the expression of IQGAP3 is upregulated in pancreatic cancer and IQGAP3-regulated pancreatic cancer cell proliferation, apoptosis, metastasis, and Cdc42 pathways.

### 4.5. Neural Wiskott–Aldrich Syndrome Protein (N-WASP)

Neural Wiskott–Aldrich syndrome protein (N-WASP), as one member of the WASP family, is widely expressed in all kinds of tissues. N-WASP has some common structural areas with WASP such as GTPase-binding domain/Cdc42 and Rac interactive binding (GBD/CRIB) regions, a proline-rich domain, and a VCA domain. N-WASP can remain inactive because of its auto-inhibiting, folded conformation. Rho GTPase, Cdc42, phosphatidylinositol 4,5-bisphosphate (PIP2), and other ligands’ interaction with N-WASP can disrupt the autoinhibited conformation and lead to activation of N-WASP. Furthermore, Arp2/3 becomes activated through binding to N-WASP that regulates actin polymerization [58].

Recent studies have shown that N-WASP may have a significant impact on tumor progression and metastasis. Hou et al. [59] have found that the expression of N-WASP is up-regulated in cervical cancer biopsies and cell lines. Additionally, N-WASP overexpression can promote migration and invasion in C33a-R cells, while N-WASP knockdown can suppress migration, invasion, and malignant behaviors in HeLa cells, probably by regulating the activity of the p38 MAPK-signaling pathway. Therefore, they concluded that N-WASP may act as an oncogene in cervical cancer. The expression of N-WASP is up-regulated in esophageal squamous cell carcinoma (ESCC) suggesting that an N-WASP assay may be helpful for the diagnosis of ESCC [60]. It has also been reported that RTVP-1 plays an important role in the migration and invasion of glioma cells through interactions with N-WASP [61]. Interestingly, the expression of N-WASP is down-regulated in breast tumors compared to normal mammary tissues [62]. Lower expression levels of N-WASP may be associated with poor patient prognosis. The function of N-WASP in breast cancer is being further explored. In addition, some N-WASP interaction partners and activators have been reported in breast cancer, such as Cdc42 interaction protein 4 and focal adhesion kinase [63,64]. In conclusion, N-WASP may play dual and cancer-specific roles in tumor progression, and further investigation will be required to elucidate these issues.

### 4.6. Phosphoinositide 3-Kinases (PI3Ks)

Phosphoinositide 3-kinases (PI3Ks), as members of the intracellular lipid kinase family, play significant roles in cell proliferation and growth, survival, polarity, apoptosis, and migration. PI3Ks are divided into three classes (I–III): Class I PI3Ks, Class II PI3Ks, and Class III PI3Ks. Class I PI3Ks are grouped into three homologous isoforms: PI3KCA, PI3KCB, and PI3KCD. They encode the highly homologous catalytic subunits p110α, p110β, and p110δ, respectively [65]. Over-activation of the PI3K/AKT signaling pathway often occurs in human cancers. On one hand, phosphatidyl inositol-4,5-bisphosphate (PIP2) is phosphorylated to phosphatidylinositol-3,4,4-trisphosphate (PIP3) by PI3K, which leads to the phosphorylation of Akt. On the other hand, phosphatidyl inositol-3,4,4-trisphosphate (PIP3) is quickly dephosphorylated to phosphatidylinositol-4,5-bisphosphate (PIP2) under the action of PTEN. The PI3K/AKT signaling pathway has been reviewed recently [66].

PI3KCB is a Class I isoform which is activated by Cdc42 and Rac1 [67]. PI3KCB plays a key role in PTEN-deficient cancers. Recently Wee et al. [68] have found that the inactivation of PIK3CB can significantly suppress tumor growth and PI3K pathway signaling in PTEN-deficient cancers. The authors also found that p110β was the key PI3K isoform that promotes abnormal proliferation and drives the activation of the PI3K pathway in PTEN-deficient cancers. In contrast, PIK3CA depletion did not affect PI3K signaling and cell growth in PTEN-deficient cancer cell lines. PIK3CB (the gene encoding p110β) promotes cancer cell proliferation and tumorigenesis in the absence of mutations, especially in wild-type PIK3CA and PTEN-deficient cancers [69,70]. Furthermore, PIK3CB mutations also have similar effects to E633K and D1067V [71,72]. In addition, the E1051K mutations may occur in lung, breast, esophageal, gastric and renal cancer cells. The p110βE1051K mutation can enhance the catalytic activity of p110β and up-regulate downstream PI3K signaling that raises the level of phosphorylated AKT and S6 compared to wild-type p110β [73]. Pridham et al. [73] found that PIK3CB/p110β plays an important role in cell survival, growth, and activation of AKT/GSK3β in glioblastoma.

## 5. Small Molecule Inhibitors of Cdc42

There is ample evidence to suggest that Cdc42 is an attractive therapeutic target in cancers. In recent years, innovative drug development has led to the introduction of novel small molecule inhibitors of Cdc42. In this section, we will discuss the discovery, molecular structure, and function of Cdc42 inhibitors in cancer therapy (Table 1).

### 5.1. ZCL278

In a recent study of the interaction of Cdc42 with intersectin (ITSN, a specific Cdc42 GEF), modeling the 3D structure of the Cdc42–ITSN complex revealed its main binding area [4,93,94]. Additionally, investigators using high-throughput screening have discovered compounds that fit into the groove on the surface of the Cdc42 structure and can play a similar role as ITSN in the binding pocket. Friesland et al. [74] have found that ZCL278 has a broad and favorable interaction with the Cdc42 residues. Their experiments also found that ZCL278 suppressed the activity of Cdc42 by directly binding to the molecule, and inhibited the migration of cultured prostate cancer cells from lines such as PC-3 but did not disrupt cell viability. ZCL278, as a Cdc42-selective small molecule inhibitor, also can suppress cellular invasion and migration in pancreatic cancer cell lines [95].

### 5.2. Secramine

Natural products, such as brefeldin A, have a significant role in the study of biological processes. Therefore, a complex natural products library was assembled of small molecules resembling galanthamine that have similar functions. A fast-acting compound found in these natural products, secramine (named because of similarities to the yeast sec mutant) was screened via a cell-based phenotypic assay and was found to be able to inhibit protein trafficking from the Golgi apparatus to the plasma membrane in monkey kidney epithelial (BS-C-1) cells and to inhibit the actin polymerization in Xenopus laevis cytoplasmic egg extract [75,76]. Secramines A and B inhibited the activation of Cdc42 through reducing the membrane association of prenylated Cdc42(GDP) in Cdc42(GDP)–RhoGDI1 in a complex fashion. However, secramine C does not share this feature. Therefore, Cdc42 and its association with RhoGDI1 is the target of secramines A and B. Secramine A suppressed the binding of Cdc42 to Golgi membranes and interfered with Golgi polarization of migrating cells.

### 5.3. CASIN

CASIN, formerly known as Pirl1, is a small molecule that acts as an inhibitor of Cdc42. Pirl1 was discovered in screening with PIP2-induced actin polymerization through a new method based on the functional suppression of chemical inhibition in vitro [78,79,96]. Peterson et al. [77] found that Pirl1 was able to inhibit nucleotide exchange on Cdc42. Furthermore, CASIN is used to treat cells in many experiments: 7 μM CASIN can suppress cytokinesis to a great extent and 20 μM can effectively kill HeLa cells within 24 h [96]. There is evidence that CASIN treatment reverts aged LT-HSC (long-term hematopoietic stem cell) to young HSCs in terms of the polar phenotype as a selective small molecule inhibitor of Cdc42 [97].

### 5.4. AZA197 and AZA1

AZA197 and AZA1 were both identified through in vitro screening of small molecule inhibitors according to modifications of NSC23766 (a specific inhibitor of GEFs binding to Rac) [98]. AZA197 can specifically inhibit Cdc42 activity through disrupting the interaction between Cdc42 and GEFs. Furthermore, Zins et al. [80] documented that AZA197 suppressed proliferation, migration, and invasion in HT-29 colon cancer cell lines and induced cancer growth in mice with human SW620 colon cancer xenografts [81]. AZA1 downregulates Rac1 and Cdc42 activity in PC-3 human prostate cancer cells and inhibits the proliferation and migration of those cells as well as decreasing formation of lamellipodia and filopodia in which Cdc42 and Rac1 play a crucial role [81]. Moreover, both AZA197 and AZA1 are able to inhibit corresponding tumor growth and increase survival in mice bearing human SW620 colon cancer xenografts [80].

### 5.5. ML141

ML141, also named CID2950007, was found to be a novel and selective Cdc42 inhibitor through high throughput screening in the Molecular Libraries Screening Center Network (MLSCN) library [82]. ML141 is a nonsteroidal anti-inflammatory drug (NSAID)-related compound and its analog, CID44216842, can inhibit Cdc42 activity by specifically blocking its GTP binding domain [84]. CID2950007 is a noncompetitive Cdc42 inhibitor. Furthermore, Hong et al. [83] reported that CID2950007 was able to inhibit the migration of OVCA429 ovarian cancer cells and decrease Cdc42 activity in breast cancer cell lines such as MCF-7, SKBR3, and MDA-MB-231. In addition, naproxen (a widely used NSAID), was also found to inhibit Cdc42 activity in a dose-dependent manner in Swiss 3T3 cells, achieving a maximum effect at 300 μM, a concentration equivalent to 10 μM ML141/CID2950007 [99].

### 5.6. PAK1-Specific Inhibitors

A series of FRAXs (486, 597, and 1036) were identified through high-throughput screening in the small molecules library using the structure-activity relationship approach. They are all small molecule inhibitors of group I PAKs (PAK1, PAK2, and PAK3) that act by directly binding to ATP. FRAX486 decreases hyperactivity and stereotypical movements and reduces audiogenic seizures in Fmr1 KO mice (knock out of fragile X mental retardation 1 gene: the mice show similar symptoms to Fragile X syndrome in humans, including seizures and hyperactivity) [85].

FRAX597 can suppress proliferation, migration, invasion, and survival in PANC-1, MiaPaCa-2, and BxPC-3 human pancreatic cancer cell lines and has a synergistic effect with gemcitabine in inhibiting the growth of pancreatic cancer in vitro and in vivo [86]. FRAX597 also inhibits SC4 cell (Schwannoma cell) proliferation in vitro through suppressing Group I PAKs and reduces tumor formation in neurofibromatosis type 2 (NF2) [87]. When combined with docetaxel, FRAX1036 synergistically inhibited microtubule function and induced apoptotic markers in MDA-MB-175breast cancer cells [88]. Although FRAXs are very good inhibitors of Group I PAKs and have good effects on some diseases and tumors, the disadvantages of FRAXs are also very obvious, and include poor bioavailability and cell-permeability as well as side effects caused by inhibition of PAK2 or PAK3. Thus, novel PAK1-specific inhibitors that have been developed recently (such as G-5555 [89], NOV-3 [90,91], and AZ137-05339 [92]) have advantages over FRAXs for development in clinical therapeutics. 

## 6. The Role of MicroRNAs in Cancer by Targeting Cdc42

MicroRNAs are small short non-coding RNA molecules with 20–24 nucleotides. They were first found in 1993. MicroRNAs are involved in many different kinds of fundamental cellular processes, including cell proliferation, cell cycle regulation, cell death, differentiation, and other functions. Most microRNAs regulate mRNA expression by binding to target mRNA 3’ untranslated regions (UTRs) through base pairing, which then results in mRNA degradation or blockage of mRNA translation [100]. MicroRNAs are short, single RNA strands that can target many mRNAs; at the same time any mRNA may have several sites allowing binding to different microRNAs. In recent years, it has been reported that microRNAs are closely related to cancer. It is now recognized that microRNAs play a significant role in carcinogenesis and progression by inducing angiogenesis, activating invasion and metastasis, reprogramming energy metabolism, evading immune destruction, and other actions [101]. Many studies have reported that Cdc42 is regulated by various microRNAs in cancer. A summary of the interactions between microRNAs and Cdc42 and its downstream effectors/adaptors is given in Table 2.

### 6.1. The Role of MicroRNAs in Lung Cancer by Targeting Cdc42

There are two types of lung cancer: small cell lung cancer (SCLC) and NSCLC. More than 80% are NSCLC, including squamous cell carcinoma, adenocarcinoma, and large cell carcinomas of three histological types. Chen et al. [122] reported that overexpression of Cdc42 is widespread in patients with primary lung cancer, and that the expression level of Cdc42 can be utilized as a prognostic marker for NSCLC metastasis. Furthermore, Li et al. [102] found that miR-29a expression levels in NSCLC tissues were significantly lower than in other non-cancer lung tissues and that miR-29a inhibited cell proliferation, migration, and invasion in these tissues by targeting Cdc42. Both nucleotides 578–584 and 997–1004 of the mRNA 3’UTR of Cdc42 have a common base 5’…UGGUGCU..., while miRNA-29a has a short base 3’…ACCACGA… [102]. Then miR-29a can bind to the mRNA of Cdc42 by base complementation and affect the function of Cdc42 in patients with NSCLC. Cdc42 also can regulate cell polarity and metastasis by affecting the formation of cellular filopodia [123]. By directly targeting the seed sequence of the Cdc42 3’UTR, miR-186 can reduce the protein expression of Cdc42 moieties and reduce metastasis of NSCLC [103]. Dacic et al. [124] reported that miRNA-137 expression was down-regulated in lung adenocarcinomas more than 20-fold compared to normal lung tissue. Furthermore, miR-137 directly targeted 3’UTR of Cdc42 and Cdk6 and negatively regulates Cdc42 and Cdk6 to inhibit cell proliferation and induce cell cycle arrest in G1 in NSCLC cells [104]. In addition, down-regulation of miR-25 contributes significantly to inhibition of cell proliferation, and to enhance chemosensitivity to cisplatin, as well as to induce cell cycle (G1) arrest in A549 cells by targeting Cdc42 [105].

### 6.2. The Role of MicroRNAs in Breast Cancer by Targeting Cdc42

The most common types of cancer in women are breast cancers, one of the major causes of death in females throughout the world [125]. According to previous research, CdcC42 induces invasiveness and metastatic activity by breast cancer cells [126]. MiR-29a can precipitate cell cycle G0/G1 arrest and suppress breast cancer cell growth through direct targeting of Cdc42 [106]. Chou et al. [107] found that miR-1 can bind to the Cdc42 3’UTR and suppress it, and thus play an important role in inducing metastasis in breast cancer. Additionally, they discovered that MALAT1 influenced the expression of Cdc42 by binding miR-1 competitively. There were ceRNA networks that co-regulated cell migration and invasion in breast cancer patients by MALALT1, miR-1 and Cdc42 [107].

### 6.3. The Role of MicroRNA-195 Targeting of Cdc42 in Esophageal Squamous Cell Carcinoma (ESCC)

Histologically, more than 90% of esophageal cancers are squamous cell carcinomas (ESCC) [127]. Expression of Cdc42 is increased in human ESCC tissues [127]. Mir-195 can suppress the expression of Cdc42 in ESCC cell lines by directly targeting its 3’UTR and inhibiting the Cdc42/ERK/Cyclin D1 signaling pathways [108]. Sun et al. [109] reported that the miR-195-Cdc42 axis was significantly involved in the progression of ESCC and that the expression of miR-195 and Cdc42 could be used as a prognostic biomarker for these cancers.

### 6.4. The Role of MicroRNAs in Gastric Cancer by Targeting Cdc42

Gastric cancer ranks as the second leading cause of cancer deaths worldwide [128]. Expression of Cdc42 is significantly increased in gastric cancer tissues and is associated with post-transcriptional regulation [112]. The expression of ectopic miR-137 can significantly suppress cell growth and influence cell apoptosis and cell cycles in gastric cancer cell lines such as AGS and MKN45 [111]. Yet the expression of miR-137 is decreased in AGS and MKN45 cell lines and negatively regulates Cdc42 by targeting its 3’UTR [111]. However, it is not clear that miR-137 functions in gastric cancer by targeting Cdc42. Cheng et al. [112] found that miR-133 inhibited the expression of Cdc42 by directly and specifically binding to its 3’UTR and then suppressing the Cdc42/PAK pathway, remarkably impeding the growth, migration and invasiveness of gastric cancer cells.

### 6.5. The Role of MicroRNAs in Colorectal Cancer by Targeting Cdc42

Colorectal cancer is one of the common malignant cancers and also one of the leading causes of death in the world today. Overexpression of Cdc42 was found in colorectal cells [129], and Cdc42 activation is thought to be one of the keys to the malignant progression of human colorectal cancer with increased cellular invasiveness [117,130]. Additionally, miR-18a can inhibit the expression level of Cdc42 through directly targeting its 3’UTR, thus decreasing cellular proliferation and migration and leading to G1/S phase arrest of the cell cycle, altering morphology and increasing apoptosis in CRC cells [113]. Liu et al. [114] found that miR-137 inhibits Cdc42 expression and the Cdc42/PAK signaling pathway by binding to the 3’UTR of Cdc42 in colorectal cancer cells, negatively regulating their invasiveness and proliferation. Studies have also suggested that miR-185 can downregulate the activity and function of RhoA and Cdc42 by targeting sites in the RhoA and Cdc42 3’UTR, which then can suppress the proliferation and invasiveness of the colorectal cancer cells [115]. The main cause of death from CRC is metastasis, with 70% of metastatic lesions occurring in the liver [131,132]. Recent studies have indicated that miR-384 can suppress the invasiveness and metastatic activity of CRC cells by downregulating expression of KRAS and Cdc42 by targeting their 3’UTR [116]. It has also been reported that miR-224 can decrease Cdc42 expression and inhibit filamentous actin-mediated cell migration by binding to the 3’UTR pf Cdc42, and thus regulate CRC metastasis [117].

### 6.6. The Role of MicroRNAs in the Downstream Signaling Pathways of Cdc42

PAK1, MLC, ERK1/2 and Cyclin D1 are the downstream effectors of Cdc42. The expression of the PAKs can be seen in all sorts of tissues and plays an essential role in promoting tumorigenesis in various human neoplasms including breast cancer [40]. PAK 1, as an oncogene in breast cancer, plays an essential role in cell proliferation, colony formation, and motility. MiR-497 can downregulate the expression of PAK1 by directly and specifically targeting its 3’UTR in breast cancer cells [118]. Other microRNAs, including miR-7, also have similar functions [119]. Ectopic expression of miR-137 decreases phosphorylation levels of PAK1, MLC and ERK1/2 and inhibits expression of Cyclin D1 in colorectal cancer cell lines [114]. Additionally, miR-195 reduces the phosphorylation levels of ERK1/2 and suppresses the expression of cyclin D1 in cell lines derived from esophageal squamous cell cancer [108].

MLK3 is another downstream effector of Cdc42. It is upregulated in several types of cancer and promotes cell proliferation, migration, invasion, and metastatic disease [133,134]. MiR-199-5p can decrease the expression of MLK3 by directly targeting its 3’UTR and it thus can suppress the MLK3/IκB/NF-κB signaling pathway in bladder urothelial carcinoma [120]. Ack1, as a downstream effector of Cdc42, has a significant role in promoting cell proliferation, migration, and invasion in a variety of human neoplasms such as hepatocellular carcinomas [135], breast cancers [21], and osteosarcomas [121]. MiR-24 suppresses Ack1 expression by directly targeting the 3’ UTR of Ack1 mRNA and significantly inhibiting the migration and invasiveness of osteosarcoma cell [121].

### 6.7. The Role of MicroRNA-224 in Hepatocellular Carcinoma by Targeting Cdc42

Hepatocyte-specific Cdc42 knockout mice showed hepatomegaly, slow growth, and chronic jaundice and frequently developed hepatocellular carcinomas at 8 months of age [136]. It has been suggested that these changes occurred because Cdc42 significantly affects the establishment of cell polarity, cell-to-cell adhesion, and spindle orientation in adherent cells. Spindle misorientation can occur with knockdown of Cdc42 without affecting cellular polarity, and these cells cannot divide and make daughter cells that will attach to the substratum [137]. Zhang et al. [110] found that overexpression of miR-224 could promote viability and migration in HCC cells, which decreased the expression of Cdc42, CDH1, and PAK2 and increased the expression of BCL-2.

## 7. Concluding Remarks and Perspectives

It is clear that Cdc42 and its signaling pathways play important roles in tumor invasion, metastasis, and proliferation. Therefore, Cdc42 is a therapeutic target for treatment of cancer. However, there are still problems that limit the utility of Cdc42 targeting. Some regulators (GAPs, GEFs, and GDIs) are not specific to Cdc42, and it does not exclusively activate all its downstream effector/adaptor proteins. For example, Rac1 can also activate ACK1, PAKs, MLK3, IQGAPs, and PI3Ks. These findings suggest that drugs targeting Cdc42 may have to be used in combination with other agents in order to get good therapeutic effects. Also, there are still some controversies over tissue-specific effects, as some studies have shown that Cdc42 can promote the progression of hepatic cancer. The prevailing explanation for this is that Cdc42 may play different roles in cancer formation depending on temporal and spatial factors, but this conclusion remains controversial. Moreover, some drugs targeting Cdc42 effector molecules have proven to be effective in vitro but to have poor therapeutic effects in experimental animal models, representing another challenge to drug development for targeted Cdc42 treatment of cancer. 

Small molecules have many advantages in the treatment of cancer, such as their being fast acting, highly specific, and having excellent pharmacokinetic properties. For example, FRAX486 can reverse symptoms after the onset of cancer; the fast-acting small molecule can pass through the blood–brain barrier (BBB) easily and remaining in the brain for 24 h [85]. In addition, downstream effector protein PAK1 (the target of FRAX597) is only expressed in the pancreatic cancer tissues, not in normal tissue; the administration of this small molecule can specifically act on cancerous tissue and reduce the side effects of damaging normal tissue [86]. 

Thus far, it appears that the therapeutic potential of small molecules to inhibit Cdc42 has been relatively underestimated, mainly due to the lack of medications specifically targeted to Cdc42. However, modern drug design and development efforts have discovered some small-molecule inhibitors of Cdc42, such as ZCL278, Secramine, CASIN, ML141, AZA197, and AZA1. Yet their specificity and cell permeability are still poor, and untoward side effects have occurred. Therefore, these small-molecule inhibitors do not approach our ultimate goal of use in hospital beds or homes for their clinical applications.

MicroRNAs play important roles in the progression and metastasis of a variety of cancers including lung cancer, breast cancer, hepatocellular carcinoma, gastric cancer, and colorectal cancer. Therefore, targeting miRNAs may offer a very effective treatment for cancer. Recent research shows that dietary phytochemicals may suppress the development and progression of cancers by modulating miRNA expression [138]. For example, sulforaphane can increase miR-140 expression and decrease miR-29a and miR-21 expression in breast cancer stem-like cells, leading to the inhibition of breast cancer stem-like cells [139,140]; epigallocatechin gallate (EGCG) can decrease miR-210 expression through upregulating hypoxia-inducible factor 1-alpha (HIF-1a) expression in lung cancer cells, which ultimately represses lung cancer cell growth [141]. Although it is reported that dietary phytochemicals play a critical role in the treatment of cancer by regulating miRNA expression, this pathway is still a long way from clinical application.

In recent years, post-transcriptional regulation by microRNAs has come to be regarded as an unconventional mechanism for regulating Cdc42, and application of competitive endogenous messenger RNA (ceRNA) has been proposed. It is known that long non-coding RNA (lncRNA) can bind directly to common microRNAs, affecting the transcription of other mRNAs. Obviously, this offers a very important strategy for the development of anti-cancer drugs. For example, MALAT1 as a type of lncRNA can promote breast cancer cell migration and invasion through competitively binding miR-1 with Cdc42 [107].

In summary, Cdc42 is a potentially valuable therapeutic target for anti-cancer therapy and offers a very promising avenue for research focused on specific Cdc42 inhibition and/or combination treatment strategies.

## Figures and Tables

**Table 1:** Selective small molecule inhibitors of Cdc42 and its downstream effectors/adaptors.

Inhibitor	Mode of Action	Reference
ZCL278	act as intersectin (ITSN) by directly binding to Cdc42	[74]
Secramine	reduce membrane association of prenylated Cdc42(GDP) in a Cdc42(GDP)–RhoGDI1 complex fashion	[75,76]
CASIN	inhibit nucleotide exchange on Cdc42	[77,78,79]
AZA197	disrupt the interaction between Cdc42 and GEFs	[80]
AZA1	disrupt the interaction between Cdc42 and GEFs	[81]
ML141	specifically block GTP binding to Cdc42	[82,83,84]
FRAXs (486, 597, and 1036)	inhibit group I PAKs (PAK1, PAK2, PAK3) by competing with ATP	[85,86,87,88]
G-5555	specific suppress PAK1 by competing with ATP	[89]
NOV-3	specific suppress PAK1 by competing with ATP	[90,91]
AZ137-05339	specific suppress PAK1 by competing with ATP	[92]

**Table 2:** (**A**) The role of microRNAs in cancer by targeting Cdc42; (**B**) The role of microRNAs in cancer by targeting downstream effectors/adaptors of Cdc42.

**A**				
**miRNA**	**Target**	**Cancer Types**	**Functional Contribution**	**Reference**
miR-29a	CDC42 3’UTR 5’…UGGUGCU…	NSCLC	inhibit proliferation, migration, invasion	[102]
miR-186	CDC42 3’UTR 5’…UUAAGAA…	NSCLC	inhibit migration and effect EMT	[103]
miR-137	CDC42 3’UTR 5’…GCAAUAA…	NSCLC		
	CDK6 3’UTR 5’…GCAAUA…	NSCLC	inhibit proliferation and induce cell cycle arrest	[104]
miR-25	CDC42 3’UTR 5’…UGCAAU…	NSCLC	reduce proliferation and induce G1 cell cycle arrest	[105]
miR-29a	CDC42 3’UTR	Breast cancer	inhibit growth through cell cycle regulation	[106]
miR-1	CDC42 3’UTR 5’…ACAUUCC…	Breast cancer	inhibit migration and invasion	[107]
miR-195	CDC42 3’UTR 5’…UGCUGCU…	ESCC	inhibit proliferation and invasion; act as a prognostic biomarker	[108,109]
miR-224	CDC42, CDH1,PAK2,BLC-2,MAPK1	Hepatocellular carcinoma	promote cell proliferation, migration, invasion; anti-apoptosis	[110]
miR-137	CDC42 3’UTR	Gastric cancer	inhibit cell cycle progression and induce apoptosis	[111]
miR-133	CDC42 3’UTR 5’…GGGGACCAG…	Gastric cancer	suppress cell growth, migration and invasion	[112]
miR-18a	CDC42 3’UTR 5’…CACCUU…	Colorectal cancer	reduce proliferation, migration; increase apoptosis; induce cell cycle arrest	[113]
miR-137	CDC42 3’UTR 5’…AAGCAAT…	Colorectal cancer	inhibit invasion, proliferation; induce cell cycle G1 arrest	[114]
miR-185	CDC42 3’UTR 5’…UGCCUUU…			
	RhoA 3’UTR 5’…UUCUCUCCA…	Colorectal cancer	inhibit proliferation, invasion; induce G0/G1 arrest	[115]
miR-384	CDC42 3’UTR 5’…UAGGAA…			
	KRAS 3’UTR 5’…UAGGAA…	Colorectal cancer	inhibit metastasis and invasive	[116]
miR-224	CDC42 3’UTR 5’…GUGACUU…			
	SMAD4 3’UTR 5’…GUGACUU…	Colorectal cancer	suppress migration and the formation of Actin Filaments	[117]
**B**				
**miRNA**	**Target**	**Cancer Types**	**Functional Contribution**	**Reference**
miR-497	PAK1	Breast cancer	suppress colonogetic ability, metastasis, tumorigenesis, invasion	[118]
miR-7	PAK1	Breast cancer	inhibit motility, invasiveness, anchorage-independent growth	[119]
miR-137	PAK1	Colorectal cancer	suppress Cdc42/pak signaling pathway	[114]
	MLC, ERK1/2			
	CyclinD1			
miR-195	ERK1/2	ESCC	suppressed Cdc42/ERK/Cyclin D1 signaling pathway	[108]
	CyclinD1			
miR-199-5p	MLK3	Bladder urothelial carcinoma	inhibit MLK3/IkB/NF-KB signaling pathway	[120]
miR-24	ACK1	Osteosarcoma	inhibit migration and invasion	[121]
